# Synthesis and Characterization of Novel Adsorbents Based on Functionalization of Graphene Oxide with Schiff Base and Reduced Schiff Base for Pesticide Removal

**DOI:** 10.3390/ma17164096

**Published:** 2024-08-18

**Authors:** Narinj Taghiyeva, Ulviyya Hasanova, Maurice Millet, Carole Gardiennet, Zarema Gakhramanova, Mushfig H. Mirzayev, Lala Gahramanli, Cuong Pham-Huu, Solmaz Aliyeva, Gunel Aliyeva, Fuad Rzayev, Eldar Gasimov, Corentin Boulogne, Haji Vahid Akhundzada

**Affiliations:** 1 CPML (UFAZ), Azerbaijan State Oil and Industry University, Azadliq Avenue, 20, Baku AZ1010, Azerbaijan; narinj.taghiyeva@etu.unistra.fr; 2 ICPEES (UMR 7515 CNRS), CNRS and University of Strasbourg, 25 Rue Becquerel, 67087 Strasbourg CEDEX 8, France; cuong.pham-huu@unistra.fr; 3 BSU (ICESCO Biomedical Materials Chair), Baku State University, Z. Khalilov Str. 23, Baku AZ1148, Azerbaijan; u.alimammad@gmail.com; 4 CRM2 UMR 7036 CNRS, Université de Lorraine, 54506 Vandoeuvre-lès-Nancy, Francecorentin.boulogne@univ-lorraine.fr (C.B.); 5 GPOGC (ASOIU), Azerbaijan State Oil and Industry University, Azadliq Avenue, 20, Baku AZ1010, Azerbaijan; zarema_q@bk.ru (Z.G.); solmaz.aliyeva@yahoo.com (S.A.); guneel.aliyeva@gmail.com (G.A.); hacivahidd@gmail.com (H.V.A.); 6 Binagadi Medical Center Named after A.D. Malikov, Azadlig 195, Baku AZ1054, Azerbaijan; musmirayev@gmail.com; 7 Nano Research Laboratory, Baku State University, Z. Khalilov Str. 23, Baku AZ1148, Azerbaijan; qahramanli.lala@mail.ru; 8 Department of Electron Microscopy of the Scientific Research Center, Azerbaijan Medical University, Nasimi Reg., S. Vurgun Str. 163, Baku AZ1078, Azerbaijan; fuad.zi@mail.ru; 9 Embryology and Histology, Azerbaijan Medical University, Nasimi Reg., S. Vurgun Str., 163, Baku AZ1078, Azerbaijan; geldar1949@gmail.com; 10 Institute of Radiation Problems of ANAS, B. Vahabzada Str. 9, Baku AZ1143, Azerbaijan

**Keywords:** graphene oxide, Schiff base, pesticide adsorption

## Abstract

Graphene oxide (GO) nanosheets were functionalized with Schiff base and reduced Schiff base. Covalent and non-covalent functionalized GO nanostructures have been tested for the removal of pesticides with different chemical structures and properties (e.g., Epoxiconazole, Dimethomorph, Cyprodinil, Chlorothalonil, Acetochlor, Trifluralin) from aqueous solutions. The structure and morphology characteristics of the prepared structures were analyzed using techniques such as solid-state nuclear magnetic resonance (SSNMR), Fourier transform infrared (FTIR) spectroscopy, X-ray diffraction (XRD), thermogravimetric analysis (TGA), scanning electron microscopy (SEM), and transmission electron microscopy (TEM). Results of the experiments showed that, although the non-covalent functionalization did not affect the adsorption properties of GO much, the covalent functionalization increased the adsorption capacity of GO against the mentioned pesticides.

## 1. Introduction

The elimination of pollutants and harmful substances can be achieved through an economical and straightforward adsorption technique. Nanomaterials are considered promising adsorbents to remove pesticides, dangerous metals, pharmaceutical contaminants, toxic organic molecules, and other pollutants from aquatic ecosystems [1]. Carbon-based nanomaterials such as carbon nanotubes, fullerenes, and graphene-based composites have attracted attention because of their huge potential in multiple applications [1,2,3].

Graphene oxide (GO) demonstrates excellent adsorption capacity for removing organic (dyes, pesticides and herbicides, bisphenol A, polycyclic aromatic hydrocarbons) and inorganic (heavy metals) pollutants from an aqueous environment. The two-dimensional nanolayer structure of GO contains a high amount of oxygen functional groups attached to the carbon lattice [2,3,4]. The presence of carboxylate, epoxy, and hydroxyl groups of GO serve for the chelating of inorganic ions. At the same time, organic low-polar compounds can be adsorbed by π-π stacking interactions with aromatic fragments of GO, and also grafted molecules that have aromatic rings in their structures [5,6,7]. At the same time, the functionalization of the GO surface by other elements could open wide spectrum applications for GO nanocomposite materials. Such doping could also introduce spacers within the GO layers, thus preventing the agglomeration process upon local reduction of the material. The functionalization of GO can be achieved through both covalent and non-covalent grafting of compounds of interest. Functionalization of GO can enhance its dispersibility, stability, and compatibility with specific solvents or matrices, expanding its potential applications. It can also introduce desired properties, such as enhanced conductivity, improved adsorption capacity, or specific chemical reactivity, depending on the nature of the functional groups or molecules attached to GO. Non-covalent functionalization involves the attachment of molecules or nanoparticles [8,9] onto the GO surface through weak non-covalent interactions, such as hydrogen bonding, electrostatic forces, π-π stacking, and van der Waals interactions [10,11]. Covalent functionalization occurs through amide or ester bond formation, in some cases by the reaction of epoxy ring opening due to nucleophilic attack [12,13].

Recently, Haque et al. [14] reported about the covalent grafting of GO with benzene-1,4-diboronic acid through the esterification reaction for CO_2_ adsorption. Cui and coworkers reported in [15] about the application of covalently functionalized GO derivatives prepared through an amidation reaction for tumor therapy application. In another study [16], the covalent modification of the GO surface with meta-toluic acid through esterification was investigated for the application of the as-synthesized composites as gas and humidity sensors. Zahirifar and co-workers [17] developed a flexible method for the functionalization of GO via the opening of the epoxide ring through a nucleophilic substitution reaction. The nucleophilic attack of octadecyl amine led to the formation of a GO/PVDF dual layer that can be used as a flat-sheet membrane for desalination. Covalent amide grafting has been used to bind amine-bearing crown ether to GO sheets for improving the adsorption properties towards metal ion sequestration [18]. The functionalization of GO with Schiff base and its metal complexes has been successfully performed in the removal of metal ions from contaminated water [19] and as catalysts in the synthesis of 1,2,3-Triazoles and 2H-Indazoles [20]. In the work reported in [21], the Schiff base was attached to GO using 3-aminopropyltriethoxysilane (APTES) as a linker. The obtained products were exploited as a Pd (II) selective probe, showing a low detection limit.

To the best of our knowledge, the data on Schiff base and reduced Schiff base functionalized GO-based adsorbents for pesticides in the literature is insufficient, unsystematized, and incomplete, pointing to the relevance of this study [2,6,22,23]. The purpose of this work is the functionalization of GO with Schiff base and reduced Schiff base for the fabrication of effective adsorbents, which will be used further in pesticide removal. The covalent functionalization of GO enhances the adsorption performance of the GO, which in turn brings cost-effective and eco-friendly materials with improved properties.

## 2. Materials and Methods

The chemicals were purchased from Sigma-Aldrich (St. Louis, MO, USA) or Ensure and used as received. All experiments were run at least twice for the sake of reproducibility.

GO was synthesized through the modified Hummers method from pure graphite. The synthesis of GO has been significantly improved by the modified Hummers method, which combines efficiency, safety, and quality to better satisfy the needs of both industrial applications and research [23]. In summary, a mixture of NaNO_3_ (5 g) and graphite (10 g) was dispersed in H_2_SO_4_ (250 mL) and swirled for 15 min in an ice bath. Then, a quantity of 35 g of KMnO_4_ was gradually introduced into the mixture while ensuring that the temperature remained below 20 °C. The mixture was agitated for 4 h after the temperature was raised to 35 °C. Next, the mixture was diluted with 450 mL of deionized water, and 2500 mL of H_2_O_2_ (3%) was added to decrease the amount of unreacted KMnO_4_. Oxidized graphite was acquired through the process of centrifugation, followed by washing with a hydrochloric acid solution diluted to a ratio of 1 part acid to 10 parts water. Subsequently, the oxidized graphite was washed with distilled water until it reached a neutral pH (20 days).

*Covalent conjugation of Schiff base (SB) (2,2’-{1,2-Ethanediylbis[nitrilo(E)methylylidene]}diphenol) and reduced Schiff base (rSB) to GO*: A total of 0.6 g of GO was dispersed in 20 mL of dimethyl formamide (DMF) (as a catalyst), followed by the addition of thionyl chloride, and the reaction was left stirring for 24 h at 70 °C. Thionyl chloride is a strong reducing agent that may transform the relatively less-reactive –COOH group of GO into the highly reactive –COCl group. Then, Schiff base was added to the reactant mixture, followed by 20 mL DMF and 1 mL triethylamine (Et3N). The temperature of the following mixture was increased to 130 °C, then stirred and refluxed for the next 72 h. The solid residue was separated by gravity filtration, followed by washing with distilled water. The residue was dried at 60 °C for 12 h to obtain the desired product [3]. The same procedure was conducted with a reduced Schiff base. In comparison to alternative approaches, this method offers a more straightforward and easily expandable procedure, although with the added difficulties of addressing environmental and safety concerns arising from the toxic properties of thionyl chloride. It is essential to consider and weigh the advantages and disadvantages when selecting the most suitable method of functionalization for certain applications. The samples obtained in this study are abbreviated as cGO-SB and cGO-rSB.

*Non-covalent conjugation of SB and rSB to GO:* GO powder was dispersed in 20 mL dichloromethane (DCM) under sonication, and Schiff base was dissolved in 20 mL DCM. After that, the two mixtures were poured together in a 250 mL round-bottom flask and refluxed for 72 h. The solid mixture was filtered and washed with DCM and was left to dry at room temperature. The same procedure was performed with rSB. The samples obtained in this study are referred to as ncGO-SB and ncGO-rSB.

*Characterization of GO and its derivatives:* The morphology and structure of GO, non-covalently functionalized ncGO-SB, ncGO-rSB, covalently functionalized GO-cGO-SB, and cGO-rSB nanostructures were characterized by Fourier transform infrared (FTIR) spectroscopy, X-ray diffraction (XRD), solid-state nuclear magnetic resonance (SSNMR), scanning electron microscopy (SEM), transmission electron microscopy (TEM) and thermogravimetric analysis (TGA) techniques.

The chemical groups in GO, ncGO-SB, ncGO-rSB, cGO-SB, and cGO-rSB nanostructures were identified using FTIR. The IR spectra were obtained using a Perkin Elmer Spectrum 100 spectrometer (PerkinElmer, Waltham, MA, USA), with wavenumbers ranging from 4000 to 400 cm^−1^.

XRD spectra were obtained using a Mini-flex 600 X-ray diffraction apparatus (Rigaku, Tokyo, Japan) from Rigaku, equipped with a Cu target (λ = 0.1540 nm), at room temperature. The system has a sealed tube X-ray source that operates at 40 kV and 15 mA. The samples were scanned in the range of 2θ = 2 to 80° using the step-scan mode.

Magic angle spinning SSNMR experiments were conducted on a Bruker AVANCE III spectrometer (Bruker, Billerica, MA, USA) operating at 300 MHz ^1^H resonance frequency (7.05 T) equipped with a 4 mm probe. The spinning frequency was set to 10 kHz. ^13^C cross-polarization (CP) spectra were performed with ^1^H and ^13^C fields of 60 kHz and 50 kHz, respectively, during a 1 ms contact time. SPINAL-64 [24] 80 kHz ^1^H decoupling was applied during 20 ms acquisition. For each spectrum, 5120 scans were recorded with a 3 s interscan delay.

SEM was utilized to acquire pictures of the produced nanostructures, employing a Zeiss Gemini SEM 500 (Jena, Germany), a SEM-FEG Schottky. The mostly used detectors are InLnese, Everhart-Thornley, and the BSD detector.

TEM, JEOL, Japan, with an accelerating voltage of 80–120 kV was used to study the morphology of the samples. All the samples were prepared by a drop-dry method on carbon-coated copper grids.

Thermal measurements were performed using the Thermal Analysis System TGA 2 (METTLER) instrument (Mettler Toledo, Columbus, OH, USA). The measurements were carried out with airflow, starting from room temperature and reaching 900 °C at a heating rate of 5 °C per minute. Before analysis, the composite was dried at 55 °C for 24 h.

Pesticide analysis: The concentration of pesticides after the adsorption experiment was determined by a gas chromatography (GC) system coupled to a DSQ II (electron impact mode (EI)) single quadrupole mass spectrometer (Thermo Scientific, Waltham, MA, USA) and an OPTIMA XLB (30 m × 0.25 mm, 0.25 µm film thickness) column from Macherey Nagel (Hoerdt, France). Injection (250 °C) was done in splitless mode (1 min), and He was used as a gas vector at a constant flow of 1 mL min^−1^. The temperatures of both the ion source and transfer line were maintained at 210 °C and 300 °C, respectively.

### Adsorption Study of Pesticide on GO and Its Derivatives

Pesticide adsorption tests were conducted using a dilution from a stock solution of pesticides (Trifluralin, Acetochlor, Chlorothalonil, Cyprodinil, Dimethomorph, Epoxiconazole) with an initial concentration of 1 gL^−1^ in distilled water. The measurements were performed as follows: 100 mg of GO, and its derivatives were suspended in 25 mL of pesticide solution (final concentration of pesticide molecules: 500 ug), and the mixtures were stirred at room temperature for 8 h. After the adsorption step, the solution was centrifugated for 15 min at 3000 rpm to completely separate the solid phase. The liquid–liquid extraction was performed with diethyl ether (25 mL total amount), and the solid phase was stored in an oven at 45 °C. After extraction, the extract was left for supernatant evaporation until 1.5 mL. Finally, 1 mL of clear solution was collected and diluted as appropriate, and the pesticide concentration was measured.

## 3. Results and Discussion

### 3.1. Synthesis and Functionalization of Graphene Oxide with SB and rSB

**Scheme 1:**
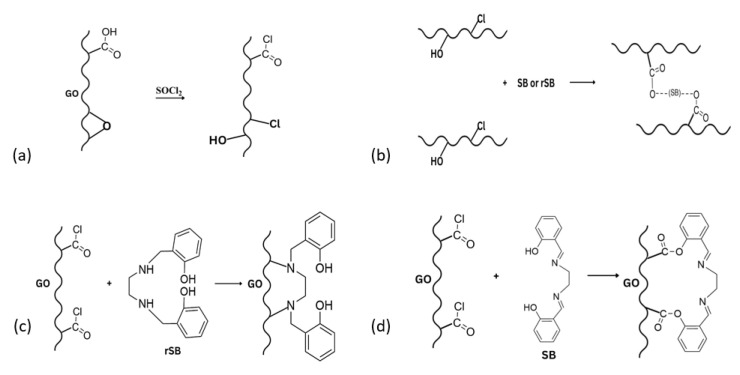
The functional groups of GO involved in covalent functionalization with SB and rSB (**a**) Activation reaction of GO; (**b**–**d**) Covalent anchoring of GO with SB and rSB.

The synthesis of GO was carried out using the modified Hummers method [23,25]. This method enables a greater output and circumvents the majority of drawbacks associated with the traditional approach. GO was chemically modified by attaching SB and rSB by covalent and non-covalent anchoring of these molecules to the oxygen-containing groups of GO. The covalent grafting of SB and rSB occurs by the activation of GO oxygen-containing groups with thionyl chloride. The activated GO interacts with SB and rSB through a nucleophilic substitution reaction, resulting in the end products known as cGO-SB and cGO-rSB. Grafting reactions are depicted in Scheme 1.

It is believed that the non-covalent functionalization of GO with both SB and rSB occurs through π-π stacking interactions (the aromatic rings of GO and SB or rSB are the active centers). Additionally, the hydroxyl and carboxyl groups of GO can form hydrogen bonds with the imine nitrogens and phenolic hydroxyl groups of SB. In the case of rSB, hydrogen bonds can be formed between the hydroxyl and carboxyl groups of GO and the amine groups and phenolic hydroxyl groups of rSB.

### 3.2. Characterization Section

#### 3.2.1. FTIR

**Figure 1:**
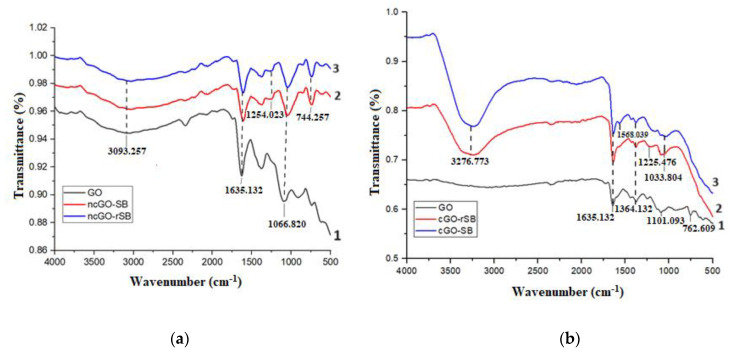
FTIR spectrum of (**a**) non-covalent conjugates (1—GO; 2—ncGO-SB; 3—ncGO-rSB); (**b**) covalent conjugates (1—GO; 2—cGO-SB; 3—cGO-rSB).

The structures of GO, ncGO-SB, ncGO-rSB, cGO-SB, and cGO-rSB conjugates have been characterized using FTIR. The FTIR analysis provided useful information regarding the structural changes of functionalized GO (Figure 1). The presence of O–H stretching on GO was confirmed by observing the broad characteristic band at a wavenumber of 3093 cm^−1^, while the stretching vibration at 1383 sm^−1^ corresponds to the C–H bond (bending) of a methyl group attached to an oxygenated group, whereas the 1086 sm^−1^ band corresponds to a C–O bond of ether [25,26]. Furthermore, the sp^2^ carbons were detected at a frequency of 1635 cm^−1^ of the C=C bond stretching. For the ncGO-SB and ncGO-rSB samples (Figure 1a—graph 2 and 3), the stretching band associated with the C–O bond is observed at a wavenumber of 1254 cm^−1^. As can be seen from Figure 1, the FTIR spectra of both ncGO-SB and ncGO-rSB differ from that of GO. These differences are most noticeable at the wavenumbers 1635 cm^−1^ and 1086 cm^−1^. After non-covalent functionalization, the observed peak at 1635 cm^−1^ shows a decrease in intensity. This can be explained by the interaction of SB and rSB with the aromatic regions of the graphene oxide through π-π stacking interactions. These interactions can alter the electron distribution in the graphene oxide, thereby affecting its vibrational modes. Additionally, the decrease in the intensity of the 1086 cm^−1^ absorption band after non-covalent functionalization can be explained by the fact that the hydroxyl groups of SB and rSB can form hydrogen bonds with the oxygen-containing groups on the graphene oxide surface. This can alter the vibrational characteristics of the ether groups.

The covalent functionalization of GO occurs via nucleophilic substitution of GO-activated oxygen-containing groups with SB and rSB molecules (Figure 1b). The peaks at a wavenumber of about 3276 cm^−1^ are attributed to O–H stretching. Stretching and contracting of the bonds in the epoxy ring, as well as aromatic C-H bending bands of pure GO that fall at a wavenumber of about 880–750 cm^−1^, disappear in the spectra of cGO-SB and cGO-rSB. Strong C–O single bond stretch between 1050–1150 cm^−1^ attributed to the epoxy group is shifted towards 1033 cm^−1^ [27]. This indicates covalent grafting through the reaction of epoxy ring opening due to nucleophilic attack. The peaks located at approximately 1640, 1225, and 1100 cm^−1^ can be attributed to vibrations of the C=O mode, an α-carbon carbonyl C–C–O stretching mode, and an O–C–C stretching mode that point to the presence of an ester bond in the grafting of SB and rSB with GO. Peaks with medium-to-weak intensity between 1300 and 1460 cm^−1^ are attributed to the antisymmetric C–N stretching vibrations. At the same time, the bands 3270 to 3500 cm^−1^ together with hydrogen-bonded association correspond to amides. The peak observed at 1568 cm^−1^ also may be attributed to the amide absorption in the cGO-rSB conjugate.

#### 3.2.2. XRD

**Figure 2:**
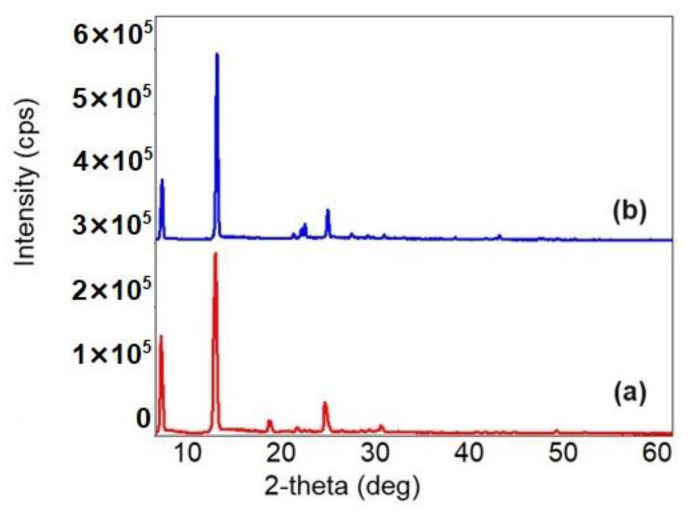
The XRD patterns of SB (**a**) and rSB (**b**).

X-ray diffraction (XRD) is a useful technique for determining the structure of polycrystalline materials. The XRD patterns of SB and rSB are presented in Figure 2.

The parameters of the peaks observed in the XRD patterns of SB and rSB are listed in Table 1. The table also lists the interplanar spacing d (nm) parameters calculated based on Bragg’s law (Equation (1)) [27] corresponding to the diffraction peaks, the crystal sizes calculated based on the Debye-Scherrer equation (Equation (2)) [28], and the lattice microstrain (ε) calculated according to Equation (3) [29]:

(1) λ=2dsinθ

where *λ* is the wavelength of X-rays (0.1542 nm), *d* represents the interplanar spacing of the crystal in nanometers (nm), and *θ* is the angle between the incident or reflected X-ray beam and the crystal plane’s surface.

(2) $$D=\frac{K\lambda }{\beta cos\theta }$$

where *K* is a constant, usually equal to 0.98, and *β* represents the full width at half maximum (FWHM) of the diffraction peak.

*ε* can be determined by the following formula by analyzing the broadening of the XRD peaks [29]:

(3) $$\varepsilon =\frac{\beta }{4tan\theta }$$

The comparison of the diffraction peaks of SB and rSB showed that diffraction peaks are observed in both samples at values close to 2θ. Only for SB, the diffraction peak at 17.029° of 2θ disappeared after reduction. Furthermore, analysis of XRD results reveals significant differences in crystallite size and strain within SB and rSB materials. The reduction process leads to the growth of larger crystallites, as evidenced by narrower FWHM values and higher Scherrer’s method crystallite size estimates for rSB, which range from 30.78 nm to 61.05 nm, compared to 24.08 nm to 38.80 nm for SB. These changes suggest that the reduction process enhances atomic mobility, promoting crystallite growth and reducing internal strain (*ε*) and lattice defects. Despite these physical changes, the crystal structure remains largely unchanged, as indicated by similar 2θ values and d-spacings between the two forms.

**Figure 3:**
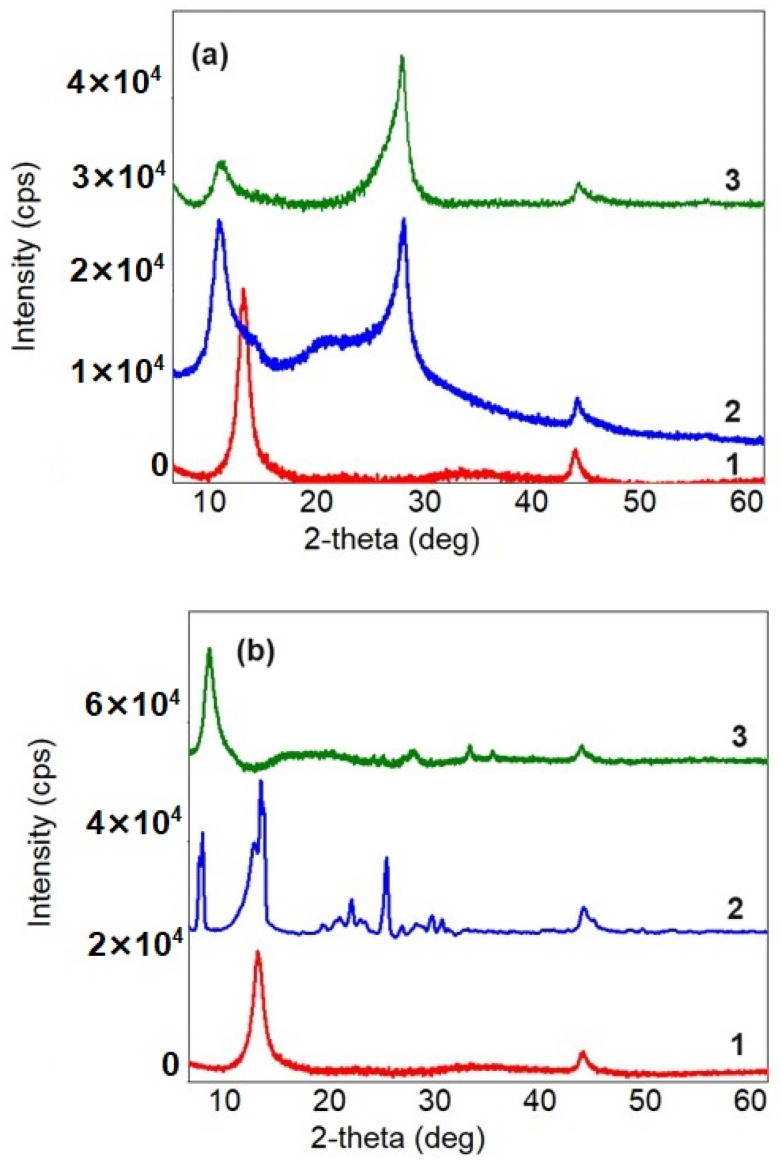
XRD patterns of (**a**) GO (1), ncGO-SB (2) and ncGO-rSB (3); (**b**) GO (1), cGO-SB (2); cGO-rSB (3).

The XRD patterns of GO and non-covalent- and covalent-bonded GO samples, namely ncGO-SB, ncGO-rSB, cGO-SB, and cGO-rSB, are shown in Figure 3.

Figure 3a,b shows that GO exhibits characteristic peaks at 11.608° and 42.162° values of 2θ. Similar results were observed by Gul et al. [30] and correspond to (001) and (100) values of hkl. After non-covalent functionalization of GO with SB and rSB, changes in characteristic peaks of GO were observed and new diffraction peaks were observed at 26.13° and 26.299 values of 2θ, respectively. Peaks observed at 26.13° and 26.299° of 2θ, respectively, for ncGO-SB and ncGO-rSB samples may be related to graphitic (002) peak due to the elimination of oxygen-containing functional groups from the surface of GO during non-covalent functionalization in the sonication environment [31]. Also, the diffraction peaks observed at 2θ values of 26.13° and 26.299° for ncGO-SB and ncGO-rSB, respectively, can be explained by the exfoliation of GO into functionalized GO with fewer layers. This phenomenon was also observed by Yin and co-workers in imidazolium-based ionic liquid-functionalized graphene oxide [32].

Table 2 lists the parameters of the (001) XRD peaks of GO, ncGO-SB, and ncGO-rSB samples, and the values of d (nm) calculated from Bragg’s law (Equation (1)) and D (nm) calculated from the Debye–Scherrer formula (Equation (2)).

It is known from the literature for GO and its derivatives that the average number of graphene layers (*N*) can be determined by dividing the crystallite size (*D*) by the interlayer distance (*d*) and considering the thickness of a graphene layer (0.1 nm) [33]:

(4) $$N=\frac{D(nm)}{d(nm)}+0.1\;nm$$

As can be seen from Table 2, during non-covalent functionalization with SB and rSB, the interlayer distance of GO increases, the crystal size decreases (*D*), and accordingly, the average number of graphene layers decreases (*N*). The reason for this is that during non-covalent functionalization, SB and rSB can combine with the π-π stacking and hydrogen interactions of GO, both on the basal and edge planes of the graphene layers. As a result of the interaction of the functionalizing molecules with the host layers, the interlayer distance increases as the layers move apart to accommodate the guest molecules. Also, functionalizing molecules interacting with the edge surfaces of the graphene sheets can cause edge expansion, due to the repulsive forces they exert on each other. Edge expansion or an increase in the interlayer distance (d) of GO leads to an increase in the length of van der Waals bonds connecting the layers and a weakening of these bonds. Since non-covalent functionalization is performed using sonication, the breaking of weakened van der Waals bonds may occur under the influence of ultrasound. This leads to the reduction of crystal size (*D*) and the reduction of graphene layer numbers (*N*) in the sample. The fact that the value of the parameter (*d*) for ncGO-rSB is smaller than that of ncGO-SB may be related to the fact that the crystal size of rSB is larger than that of SB (as can be seen from the XRD results (Table 1)). Thus, entering between two neighboring edge surfaces of GO and interacting with them may be somewhat more difficult for rSB, with a large crystal size, than for SB.

After covalent functionalization of GO with SB and rSB, the peak of 2θ at 11.608° was observed for cGO-SB at 6.324° and cGO-rSB at 6.76°. It shows that the Bragg’s law d-spacing of GO increases from 0.76 nm to 1.40 nm for cGO-SB and 1.31 nm for cGO-rSB. It can be seen from the results that the increase of d-spacing during covalent functionalization was greater than that of non-covalent functionalization. In the diffraction patterns of cGO-SB and cGO-rSB, new peaks characterizing structural changes during covalent functionalization are observed in the interval of 10–40° of 2θ. Additionally, the (100) diffraction peak observed at a 2θ value of 42.162° characteristic of GO was observed at 42.51° in cGO-SB and 42.28° in cGO-rSB. The observed diffraction peaks corresponding to the graphitic peak (002) at 26.6° for cGO-SB and 26.28° for cGO-rSB may correspond to a partial reduction of GO upon functionalization [34,35], causing a decrease in the number of functional groups on the GO surface and the appearance of a (002) diffraction peak of typical disordered carbon materials. A similar phenomenon was also observed in covalent functionalized GO with aromatic and non-aromatic amines, such as dibenzylamine, p-phenylenediamine, diisopropylamine, piperidine [34,35], hexamethylene diamine, and liquid NH_3_ [34].

### 3.3. Solid-State NMR Analysis

**Figure 4:**
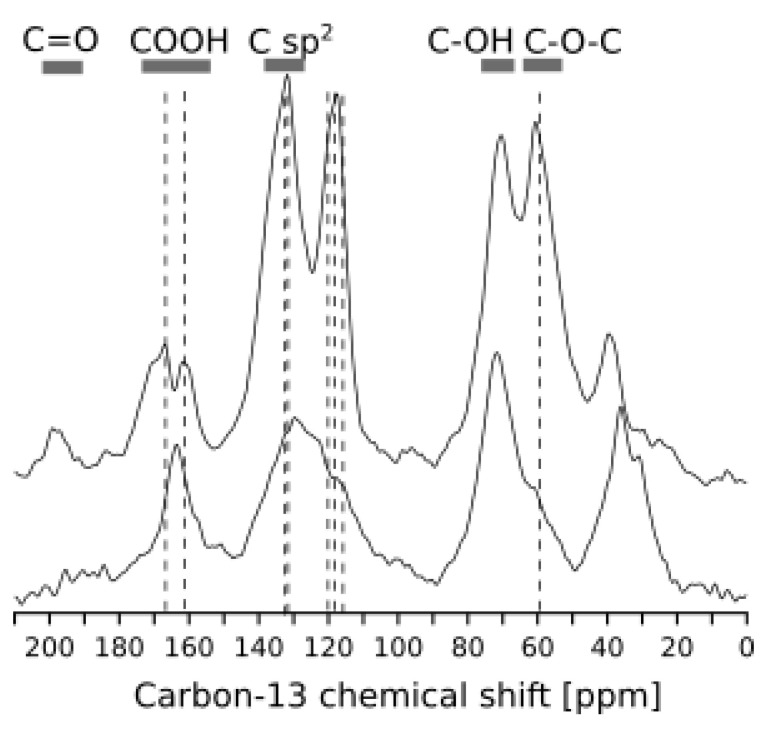
^13^C CP NMR spectra of fGO covalently (bottom) or non-covalently (top) functionalized with SB. Dotted vertical lines indicate signals of pure SB. Thick horizontal lines are drawn in regions corresponding to pure fGO signals [30].

The ^13^C CP NMR spectra presented in Figure 4 show typical features of graphene oxide, together with signals expected from SB. The relative intensity of the peak at ~60 ppm assigned to fGO epoxide groups is lower in the sample where SB is covalently attached to fGO, pointing to a functionalization via ring opening of fGO epoxides, as visible in Figure 4. The new peak appearing at ~30 ppm in covalent samples could correspond to the methylene carbon alpha to the nitrogen atom after the epoxy ring opening. Our NMR data neither confirms nor excludes covalent functionalization through amidation.

#### 3.3.1. SEM

**Figure 5:**
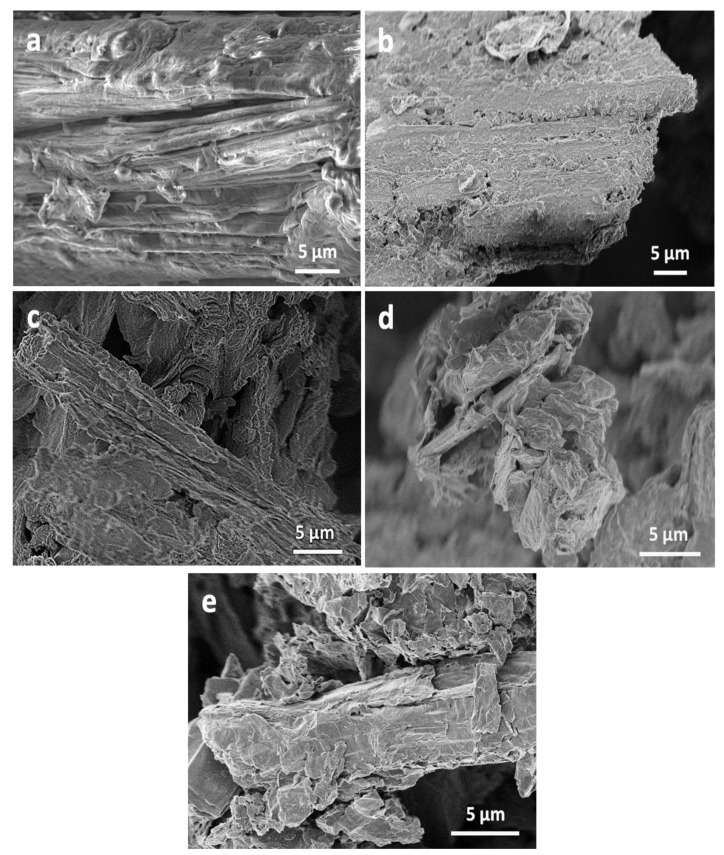
SEM images of GO and its derivatives: (**a**) GO; (**b**) ncGO-SB; (**c**) ncGO-rSB; (**d**) cGO-SB; (**e**) cGO-rSB.

The morphology of pure GO and functionalized GO samples was studied using SEM, and the results are shown in Figure 5. It can be seen from the figure that pure GO consists of densely packed sheets (GO nanosheets aggregate by forming networks of hydrogen bonds and van der Waals forces between their layers [32]) with a smooth surface and rounded or irregular edges [36,37], and this morphology is different from ncGO-SB, ncGO-rSB, cGO-SB, and cGO-rSB. Crushed flakes with wrinkles in random directions, some folded regions, and loosened lamellar structures resulting in irregular structures are observed in the functionalized samples and are more prominent in the covalently functionalized samples. Specifically, the surface roughness of ncGO-SB and ncGO-rSB is less than that of cGO-SB and cGO-rSB. These results indicate the functionalization-induced dispersion of SB and rSB on the GO surface with both covalent and non-covalent bonds [38]. Careful examination of the SEM images of the samples reveals that the distribution and attachment of the functionalizing agents (SB and rSB) between the GO sheets and on the surface of the GO result in a decrease in the stacking level of GO layers. As a result, the interlayer distance expands [39]. Similar observations were made by Alkhouzaam and co-workers for polydopamine functionalized graphene oxide [39].

#### 3.3.2. TEM

**Figure 6:**
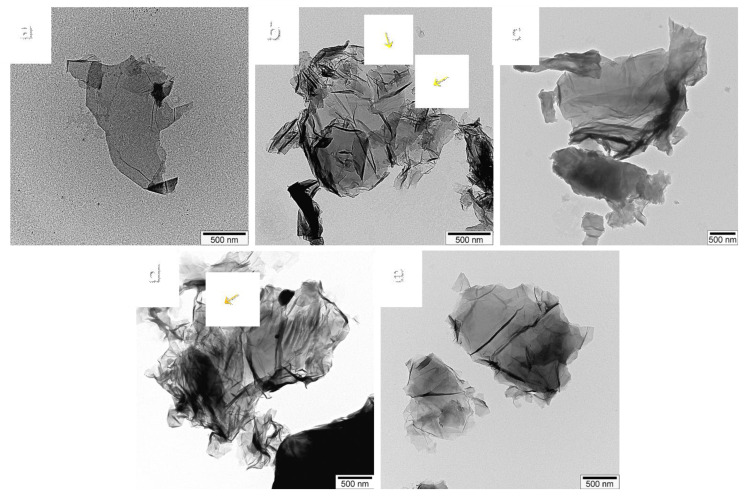
TEM images of (**a**) GO; (**b**) ncGO-SB; (**c**) ncGO-rSB; (**d**) cGO-SB; (**e**) cGO-rSB.

Morphological characterization of the samples was carried out using TEM research. Bright-field TEM images of GO, ncGO-SB, ncGO-rSB, cGO-SB, and cGO-rSB samples are shown in Figure 6. From the TEM image of GO (Figure 6a), it can be seen that GO has a clear layered structure, and the surface is clean and smooth. Due to its small atomic thickness, it exhibits a partially transparent surface. As can be seen from the TEM image (Figure 6a), there are oxygen-containing functional groups on the surface of GO, and the strong interaction between these groups can easily cause crumps and folds to form on the GO surface [40]. After both non-covalent and covalent functionalization, many opaque regions are observed on the surface of ncGO-SB, ncGO-rSB, cGO-SB, and cGO-SB samples. These phenomena indicate that SB and rSB are successfully attached to the GO surface through both non-covalent and covalent bonds, and these molecules weaken the electron transfer capability of the sample [41]. Similar phenomena were also observed for non-covalently functionalized GO with hyperbranched polyesters with terminal carboxyl [42] and with phenyl polyhedral oligomeric silsesquioxane, and covalently functionalized GO with polyglycerol [43]. It can be seen from Figure 6b–e that the dark colors are more abundant on the edge surfaces of GO than on the basal surfaces, indicating that the molecules are more attached to the edge surfaces.

From Figure 6, it can be seen that the bending and shrinkage of the nanosheets increased after both non-covalent and covalent functionalization. This is because there are more hydrogen bonds between the functionalizing molecules.

#### 3.3.3. TGA

**Figure 7:**
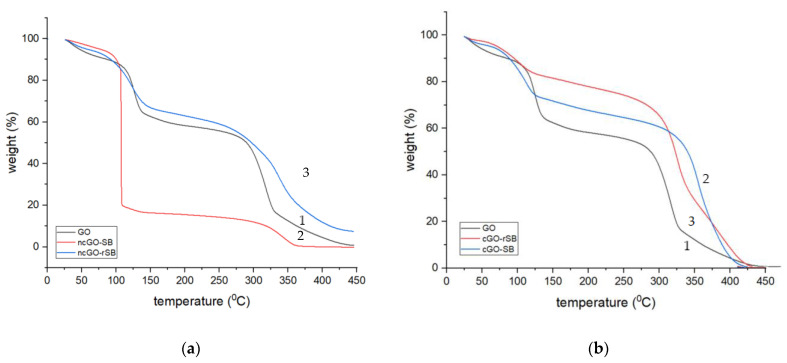
TGA curves of (**a**) non-covalent conjugates GO (1—GO; 2—ncGO-SB; 3—ncGO-rSB); (**b**) covalent conjugates GO (1—GO; 2—cGO-SB; 3—cGO-rSB).

To confirm the success of the functionalization of GO with SB and rSB, all samples were analyzed using thermogravimetric analysis (TGA). The TGA results of the samples are depicted in Figure 7a,b.

The decrease in mass of all samples at around 100 °C is attributed to the evaporation of moisture and water molecules that were adsorbed. The π-stacked arrangement of graphene oxide (GO) allows the retention of some water molecules, leading to a weight loss of around 7.3 wt. % up to 110 °C [44]. According to the results, GO has two more primary degradation phases occurring between 110–450 °C. A significant decrease in weight, approximately 30%, occurring between 110 and 210 °C is attributed to the formation of CO, CO_2_, and H_2_O from the most unstable functional groups [45]. The gradual decrease in mass between 210 and 450 °C is caused by the breakdown of more-durable oxygen groups and aligns with the observed weight loss of approximately 18 wt. %. As previously reported, Schiff base and reduced Schiff base simultaneously reduce, functionalize, and stitch on GO nanosheets [46]. Stitching occurs due to the existence of two amine (–NH–) and imine (=N–) groups on both sides of the SB and rSB moiety. When two –NH– and =N– functional groups react with the epoxide carbon/carboxylic moieties of two distinct GO sheets, it contributes to the formation of a stitched structure. Results indicate that the storage of water molecules in a π-stacked structure is significantly reduced and a mass loss of approximately 4.6 wt. % is observed after the interaction of GO with SB. The second stage of degradation shifts from 165 to 196 °C, with a weight loss of around 12 wt. %. SB and rSB considerably decrease the oxygen-containing groups on GO nanosheets or react with unstable groups, resulting in increased thermal stability. Instead of degrading at 266 °C due to amine group degradation, a distinct degradation occurs at 280 °C, followed by a gradual degradation with a maximum degradation temperature of 312 °C that corresponds to the degradation of SB and rSB aromatic groups attached to GO. It is also noteworthy that attaching SB and rSB onto nanosheets using covalence enhances thermal stability above 310 °C and increases mass loss. In cGO-SB and cGO-rSB samples, the maximum decomposition temperature increases to 387 °C and 411 °C, respectively.

### 3.4. GO and Its Derivatives Tested for Pesticide Adsorption

GO has a high adsorption capacity for water-soluble contaminants, particularly positively charged moieties, dyes, and metal ions [47,48], and slight changes in conditions can significantly affect the results. The mechanism of action may involve many physicochemical processes, such as complexation, physical adsorption, precipitation, ion-exchange, and electrostatic interaction [18,48].

For our study, several pesticides were selected (Trifluralin, Acetochlor, Chlorothalonil, Cyprodinil, Dimethomorph, and Epoxiconazole) as representative pollutants to analyze their adsorption on GO and its covalently functionalized derivatives. The percentage of removal (% R) was calculated according to Equation (5):

(5) $$\%R=\frac{C_{i}-C_{f}}{C_{i}}*100\%$$

where $C_{i}$ and $C_{f}$ are the pesticide concentrations initially and after the adsorption processes (mg/L). Table 3 displays the obtained results on the different samples.

It is worth mentioning that batch experiments with GO, ncGO-SB, ncGO-rSB, cGO-SB, and cGO-rSB ran under the same conditions for comparative purposes. However, no reliable results could be obtained from the experiment with non-covalently functionalized GO derivatives. The water was polluted with Schiff base and reduced Schiff base instead.

According to the optimized results, the values for GO are less than cGO-SB and cGO-rSB. The remarkable increase in the percentage of removal can be explained by the increase in porosity and active surface area of cGO-SB and cGO-rSB.

## 4. Conclusions

In conclusion, both covalent and non-covalent functionalization of GO with SB and rSB were successfully performed and thoroughly analyzed using various methods such as SSNMR, FTIR, XRD, TGA, SEM, and TEM. FTIR results indicated that non-covalent functionalization occurs via π-π stacking and hydrogen bonding, while covalent functionalization happens through nucleophilic substitution of oxygen-containing groups. XRD studies revealed an increase in the d-spacing of GO after functionalization, and SEM and TEM analyses showed morphological changes in GO. TGA analysis demonstrated the enhanced thermal stability of GO upon covalent grafting of SB and rSB, with stability improving above 310 °C.

In practical applications, the functionalized GO samples were tested for the adsorption of various pesticides from aqueous solutions. While non-covalently functionalized GO (ncGO-SB and ncGO-rSB) samples did not show significant adsorption capabilities, covalently functionalized GO (cGO-SB and cGO-rSB) samples exhibited significantly improved adsorption properties. This enhancement is attributed to the increased active centers resulting from functionalization. Notably, the adsorption efficiency for pesticides such as Epoxiconazole, Dimethomorph, Cyprodinil, Chlorothalonil, Acetochlor, and Trifluralin dramatically increased. For example, the adsorption of Chlorothalonil increased from 9% with GO to 90% with cGO-SB and cGO-rSB. Thus, covalently functionalized GO samples (cGO-SB and cGO-rSB) demonstrated superior efficiency in pesticide adsorption compared to non-functionalized GO, making them promising materials for environmental cleanup applications.

## Figures and Tables

**Table 1:** Parameters of peaks observed in XRD patterns of SB and rSB.

SB					rSB				
2θ	β	d, nm	Scherrer’s Method D (nm)	ε	2θ	β	d, nm	Scherrer’s Method D (nm)	ε
11.396	0.342	0.78	24.39	0.01496	11.5464	0.2337	1.57	35.70	0.01009
17.029	0.271	0.52	30.97	0.00790	-	-	-	-	-
20.058	0.358	0.44	23.55	0.00883	20.875	0.190	0.43	44.42	0.00450
22.945	0.29	0.39	29.21	0.00623	23.313	0.234	0.38	36.22	0.00495
26.91	0.22	0.33	38.80	0.00401	25.840	0.186	0.34	45.79	0.00354
27.71	0.35	0.32	24.43	0.00619	27.55	0.14	0.32	61.05	0.00249
28.994	0.356	0.31	24.08	0.00601	29.311	0.213	0.30	40.28	0.00355
41.16	0.27	0.22	32.84	0.00314	41.5	0.188	0.22	47.22	0.00217
47.601	0.324	0.19	28	0.00321	47.713	0.255	0.19	35.59	0.00252

**Table 2:** Parameters of (001) peaks and *d* (nm), *D* (nm), and *N* values of GO, ncGO-SB and ncGO-rSB.

Sample	(hkl)	2θ	β	d (nm)	Scherrer’s Method D (nm)	Number of Graphene Layers (*N*) per Domain
GO	(001)	11.608	1.228	0.76	6.84	~9
ncGO-SB	(001)	9.12	5.97	0.97	1.39	~2
ncGO-rSB	(001)	9.61	1.69	0.92	4.93	~5

**Table 3:** Removal percentage (%R) for all batches, including a ±15% uncertainty, of pesticides by GO, cGO-SB, and cGO-rSB.

Structure	Name of Pesticides/Adsorbents	GO	cGO-rSB	cGO-SB
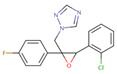	Epoxiconazole	65 ± 9	82 ± 12	82 ± 12
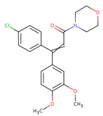	Dimethomorph	79 ± 11	82 ± 12	82 ± 12
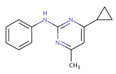	Cyprodinil	88 ± 13	91 ± 14	91 ± 14
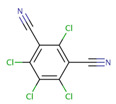	Chlorothalonil	9 ± 1	90 ± 13	90 ± 13
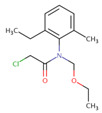	Acetochlor	45 ± 7	86 ± 13	94 ± 14
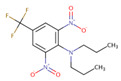	Trifluralin	70 ± 10	94 ± 14	96 ± 14

## Data Availability

The original contributions presented in the study are included in the article, further inquiries can be directed to the corresponding author.
